# Non-invasive vagus nerve stimulation is associated with the reduction in persistent post-concussion symptoms: an observational study

**DOI:** 10.3389/fneur.2025.1642034

**Published:** 2025-08-26

**Authors:** Michael Ament, Emily Leonard, Peter S. Staats, Norianne T. Ingram

**Affiliations:** ^1^Cherry Creek Neurology, Denver, CO, United States; ^2^Vagus Nerve Society, Atlantic Beach, FL, United States; ^3^electroCore, Inc., Rockaway, NJ, United States

**Keywords:** traumatic brain injury, concussion, non-invasive vagus nerve stimulation (nVNS), post-concussion symptoms, persistent symptoms, neuroinflammation

## Abstract

**Introduction:**

Traumatic brain injury (TBI) remains a major public health challenge, with mild TBI (mTBI) frequently resulting in persistent cognitive, affective, and somatic symptoms. Non-invasive vagus nerve stimulation (nVNS) has demonstrated potential in reducing neuroinflammation and promoting recovery in preclinical TBI models. This retrospective, observational study assessed the impact of adjunctive nVNS on postconcussive symptoms in routine clinical practice.

**Methods:**

We conducted a single-center observational cohort study consisting of 102 patients with mTBI who received adjunctive nVNS as part of standard care. Symptom severity was measured using the Neurobehavioral Symptom Inventory (NSI) at baseline and approximately 112 days post-treatment initiation. The primary outcome was the change in NSI symptom scores. Secondary analyses explored associations between baseline symptom severity, treatment response, and secondary clinical measures. Safety data were collected throughout the study period.

**Results:**

In this patient cohort, 16 of 22 NSI symptom domains showed significant improvement after three months of treatment with adjunctive nVNS. The most notable reductions were observed for post-traumatic headache (−0.79 ± 1.19; *p* = 1.97 × 10^−8^), difficulty concentrating (−0.59 ± 1.25; *p* = 1.79 × 10^−5^), dizziness (−0.47 ± 1.14; *p* = 7.11 × 10^−5^), and depression/sadness (−0.47 ± 1.12; *p* = 9.09 × 10^−5^). Approximately onethird (34%) of patients met responder criteria (≥30% reduction) for at least half of the assessed symptoms, indicating broad and clinically meaningful symptom relief. The length of the interval between injury and treatment initiation did not significantly influence baseline symptom severity or the magnitude of improvement. No devicerelated adverse events were reported.

**Discussion:**

In a naturalistic clinical setting, adjunctive nVNS was associated with significant reductions in post-concussive symptom severity across cognitive, affective, somatic, and vestibular domains in patients with mTBI. These findings support the use of nVNS as a practical, safe, and effective intervention for persisting symptoms due to mTBI. Further prospective, controlled studies are warranted to validate these observations and elucidate underlying mechanisms.

## Introduction

Traumatic brain injury (TBI) represents a critical public health concern, affecting an estimated 5.3 million Americans and contributing to an annual economic burden of approximately $60 billion (1, 2). The burden falls disproportionately on young adults (under 45), who often experience long-term disability and social withdrawal during their most productive years (3, 4). Even mild TBI (mTBI) can result in persistent cognitive, emotional, and somatic symptoms that disrupt daily functioning and are often resistant to standard rehabilitative care (5, 6). Secondary injury mechanisms—particularly neuroinflammation—are increasingly recognized as key contributors to long-term impairment, with elevated cytokines such as interleukin-1β (IL-1β) implicated in synaptic dysfunction and progressive neural damage (7, 8). Therapeutic strategies targeting these inflammatory cascades are gaining traction; vagus nerve stimulation, both invasive and non-invasive, has been shown to modulate IL-1β signaling, reduce neuroinflammation, and improve functional outcomes in models of TBI and CNS injury (9–13).

Building on the evidence that vagus nerve stimulation modulates injury cascades, non-invasive vagus nerve stimulation (nVNS) specifically has garnered increasing attention as a safer, more accessible intervention capable of targeting multiple components of the TBI injury cascade (14, 15). Preclinical studies demonstrate that nVNS can suppress IL-1β activation in injured neurons, reduce lesion volume, and improve behavioral performance in both closed-head and contusion models of TBI (11, 12, 16). These effects are thought to arise through modulation of both central and peripheral inflammatory pathways, including cholinergic anti-inflammatory circuits and afferent projections to brainstem nuclei such as the nucleus tractus solitarius and locus coeruleus (10, 17, 18). Additional mechanisms of nVNS include reduced glutamate-mediated excitotoxicity, improved blood–brain barrier stability, and enhanced neurochemical support for plasticity and recovery (4, 9, 19, 20). Together, these findings provide a strong biological rationale for exploring nVNS as a tool to mitigate secondary injury processes and enhance functional outcomes in patients with TBI.

Despite decades of clinical research, effective treatments for TBI-related cognitive dysfunction remain elusive (21). Historically, TBI has often been under-recognized as a chronic disability, particularly in cases of mTBI where symptoms can persist despite normal imaging and apparent resolution of the acute injury (5). These lingering symptoms—encompassing persistent cognitive, emotional, and physical impairments—affect a substantial subset of patients with mTBI and contribute to long-term reductions in quality of life (5).

Current treatment options—such as pharmacologic agents (e.g., amantadine, methylphenidate) and cognitive rehabilitation programs—offer limited efficacy and are often constrained by patient-specific factors such as motivation, severity, and access to care (6, 22). In this context, nVNS emerges as a mechanistically targeted, well-tolerated, and easily deployable intervention with the potential to address a major therapeutic gap in both acute and chronic phases of TBI recovery (10, 12, 14, 16).

Building on this foundational evidence, we conducted a retrospective analysis of 102 patients with mTBI to characterize symptom trajectories associated with adjunctive nVNS administration in a clinical setting. Given the absence of approved pharmacological or device-based treatments for post-concussive dysfunction, exploration of novel interventions such as nVNS is (14, 21). By leveraging clinical data from an observational cohort, we sought to assess whether nVNS may offer practical and mechanistic advantages in a population with heterogeneous symptom presentation (6, 14, 23).

## Methods

### Study design

This retrospective, single-center observational cohort study evaluated the effects of adjunctive non-invasive vagus nerve stimulation (nVNS) on post-concussive symptoms in patients with mild traumatic brain injury. Data were collected from routine clinical care at Cherry Creek Neurology (Denver and Colorado Springs, CO) between February 2021 and September 2024, as part of ongoing quality assurance. All patients provided written informed consent for research use of their de-identified clinical and survey data. The study was conducted under an IRB-approved protocol (IRB# 202301118, University of Florida). nVNS was prescribed at clinician discretion as part of standard care; no experimental interventions were administered.

As part of standard intake, all patients completed the Neurobehavioral Symptom Inventory (NSI), a validated 22-item instrument that assesses cognitive, affective, somatic, and vestibular symptoms on a 0–4 scale, with 4 representing the most severe symptom burden (Table 1). Mean scores are reported for individual symptoms and composite domains. Follow-up NSI assessments were collected on an average 112 days after treatment initiation to evaluate symptom change over time. Both baseline and follow-up scores were required to be included into the main analysis cohort (*n* = 102).

**Table 1:** NSI symptom domains and average severity at baseline and after treatment with adjunctive nVNS.

NSI domains	Baseline NSI Mean	Baseline NSI STD	Follow-up NSI mean	Follow-up NSI STD	*p*-value
Dizziness	1.99	1.04	1.52	1.00	0.00007
Loss of Balance	1.78	1.18	1.33	0.97	0.00014
Poor Coordination	1.82	1.08	1.50	1.03	0.00205
Post-Traumatic Headaches	2.91	0.95	2.12	1.09	1.97 × 10^−8^
Nausea	1.49	1.22	0.99	1.12	0.00012
Vision Problems	1.75	1.23	1.66	1.28	0.35021
Light Sensitivity	2.05	1.25	1.70	1.15	0.00133
Difficulty Hearing	1.20	1.11	1.00	1.08	0.09316
Sensitivity to Noise	1.94	1.25	1.52	1.16	0.00083
Numbness/Tingling	1.62	1.28	1.36	1.24	0.07110
Altered Taste/Smell	0.66	1.07	0.50	0.96	0.10391
Appetite	1.37	1.23	1.07	1.17	0.00663
Poor Concentration	2.59	1.15	2.00	1.16	0.00002
Forgetfulness	2.62	1.11	2.20	1.20	0.00085
Decision Making	2.16	1.18	1.75	1.18	0.00276
Slowed Thinking	2.53	1.14	2.09	1.24	0.00077
Fatigue	2.66	1.18	2.19	1.19	0.00013
Falling Asleep	2.47	1.23	1.97	1.18	0.00033
Anxious/Tense	2.51	1.18	2.09	1.13	0.00110
Depressed Sad	2.11	1.25	1.64	1.22	0.00009
Irritability	2.39	1.19	1.87	1.23	0.00013
Easily Overwhelmed	2.55	1.18	2.07	1.25	0.00027
Vestibular Score	5.75	2.87	4.52	2.76	0.00012
Somatic Score	12.59	5.36	9.92	5.77	1.06 × 10^−6^
Cognitive Score	10.16	3.88	8.13	4.37	0.00005
Affective Score	14.81	5.62	11.58	6.41	4.43 × 10^−6^
Total Score	45.80	16.20	36.08	18.70	2.03 × 10^−7^

NSI symptom domains (rows 1–22) and the average patient scores (*n* = 102) with standard deviations. Wilcoxon signed-rank was used to compare means. Significant differences were found for *p* < 0.00227. Rows 23-26 show composite scores based select symptoms listed above. The last row shows the “Total Score” and averages all 22 NSI symptoms.

### Population

A total of 175 patients with a clinical diagnosis of mTBI were screened. Of these, 102 patients had both baseline and follow-up NSI data indicating a drop-out rate of 73 patients (42%). Final analyses were conducted on the 102 patients with both NSI surveys. This cohort consisted of 66 male (64.7%) and 36 female (35.3%) participants, with a mean age of 40.7 ± 14.6 years at intake. The average time from injury to treatment initiation was 171 ± 243 days. Eleven percent of patients reported symptom persistence for more than 1 year prior to starting nVNS.

Patients were not considered for nVNS therapy if they had an implanted pacemaker or defibrillator, implanted medical devices other than orthopedic hardware, a history of a cervical spinal fusion or surgery at or above C4, in the past had a surgery to the anterior neck (ex. radical neck dissection for cancer or carotid artery surgery) or if the patient was at the time pregnant.

As this study was retrospective, the patient cohort included was highly heterogenous and comorbidities were not tracked. Patients were included based on clinical diagnosis of mTBI with persistent symptoms post-concussion and the completion of baseline and follow-up NSI assessments. While there were not strict inclusion and exclusion criteria, patients with more severe headache and psychological NSI scores typically had nVNS included in their treatment protocols. Anxiety and PTSD symptoms were assessed during each patient’s initial visit, and if present, they were considered for nVNS treatment. Alternative options for patients with depression, anxiety, and/or PTSD symptoms included counseling and medication.

### Treatment protocol

At the time of intake, all patients completed the NSI to establish a baseline measure of post-concussive symptoms. Additional self-report instruments—including the Pain Catastrophizing Scale (PCS), the Generalized Anxiety Disorder 7-item scale (GAD-7), the PTSD Checklist for DSM-5 (PCL-5), the Patient Health Questionnaires for depression (PHQ-9), and for somatic symptoms (PHQ-15)—were administered based on clinical discretion. Following intake, patients were prescribed gammaCore™ (electroCore, Inc.), a cervical, non-invasive vagus nerve stimulator, as an adjunctive treatment alongside standard clinical management. The nVNS device runs for 2 min at a time and generates a bursting sinusoidal wave that is designed to stimulate the vagus nerve at 25Hz. It outputs a maximum of 60 milliamps and 24 volts.

Once patients were prescribed nVNS, they were trained to use the device during an in-office appointment. Patients were instructed to position the device over their cervical pulse point and turn the device up to an intensity which elicited a lip-pull or twitch. Instructions were to use the device daily: 2-min stimulations twice in the morning and twice in the evening (at least 4 total sessions) as well as any time that the patient felt symptomatic. During the in-office training, patients scheduled a 2-week follow up appointment and received daily texts during those 2 weeks with reminders to use the device. At the follow-up appointment, patients would answer questions about symptom improvement, demonstrate their use of the device, and work through any problems related to using the device (e.g., device placement, intensity).

Follow-up NSI surveys were collected on average 112 ± 77 days after treatment initiation to evaluate changes in symptom severity.

nVNS was given adjunctively to standard of care, which was patient specific. Briefly, patients with clinically confirmed or probably concussion were evaluated on symptom clusters: headache, vision, vestibular/autonomic, auditory, cognitive, psychological, or sleep issues. Appropriate neurological and physical examinations were administered based on symptom complaints. Patients were then referred to specific therapies targeted to identify abnormalities/symptoms. Patients would meet with physician/therapists at regular intervals, every 4–8 weeks, to review test results and response to treatments, repeat appropriate examinations, and discuss options and treatment plans.

### Outcome measures

The primary outcome was change in symptom severity as measured by NSI total and item scores from baseline to follow-up. Secondary analyses included correlations between NSI outcomes and scores from optional supplementary instruments (PCS, GAD-7, PCL-5, PHQ-9, PHQ-15), when available. In addition, we examined whether the elapsed time between injury and treatment initiation was associated with either baseline symptom severity or the magnitude of symptom improvement following nVNS treatment. Adherence was tracked.

### Statistical methods

Paired comparisons of mean NSI scores at baseline and follow-up were conducted using Wilcoxon signed-rank tests. To control for multiple comparisons across 22 NSI symptoms, a Bonferroni correction was applied, setting the significance threshold at *p* < 0.00227. Between-group analyses (e.g., stratified by injury age) were performed using one-way ANOVA with Scheffé’s *post hoc* testing or Mann–Whitney U-tests. For these tests statistical significance was set at *p* < 0.05. Pearson correlation analyses and linear regressions were conducted to explore relationships between baseline symptom burden and treatment response, as well as between NSI scores and secondary survey instruments. Analyses were performed using custom scripts written in Matlab.

### Safety assessments

No device-related adverse events were reported during the 3-month treatment period.

## Results

### Demographics

Data were collected from 175 patients between February 2021 and September 2024. All patients completed the NSI at intake. Additional surveys—including the PCS, GAD-7, and PCL-5—were administered when clinically indicated. Shortly after intake, patients were prescribed a non-invasive vagus nerve stimulator (gammaCore™, ElectroCore, Inc.) for a treatment duration of 3 months. Follow-up NSI assessments were completed approximately 112 days after baseline.

Of the 175 patients, 102 had complete baseline and follow-up NSI data and were included in the final analysis. This cohort included 66 males (64.7%) and 36 females (35.3%), with a mean age at intake of 40.7 ± 14.6 years. The average time from injury to treatment initiation was 171 ± 243 days. Eleven percent of patients were treated for symptoms persisting for more than 1 year. The mean baseline NSI score was 2.05 ± 0.56. On average, patients used the device on 56 of the 90 study days, reflecting a 62% compliance rate.

A retrospective attempt was made to evaluate a separate cohort of patients who did not receive nVNS. However, their mean baseline NSI scores (1.49 ± 0.48) were substantially lower than those in the nVNS-treated cohort, suggesting a systematically different population. No further comparisons were performed.

### Adjunctive nVNS facilitates post-concussion recovery across diverse symptom presentation

Of the 102 subjects in this study, 51% of them had experienced symptoms for 6 months or more. The most severe symptoms patients experienced when seeking treatment were: *post-traumatic headaches* (*NSI: 2.91* ± 0.95), *fatigue* (*NSI: 2.66* ± 1.18), *forgetfulness* (*NSI: 2.62* ± 1.11), *difficulty concentrating* (*NSI: 2.59* ± 1.15), and feeling *easily overwhelmed* (*NSI: 2.55* ± 1.18) demonstrating a board presentation symptoms in multiple domains (e.g., Cognitive, Somatic, Affective; See Table 1).

**Figure 1:**
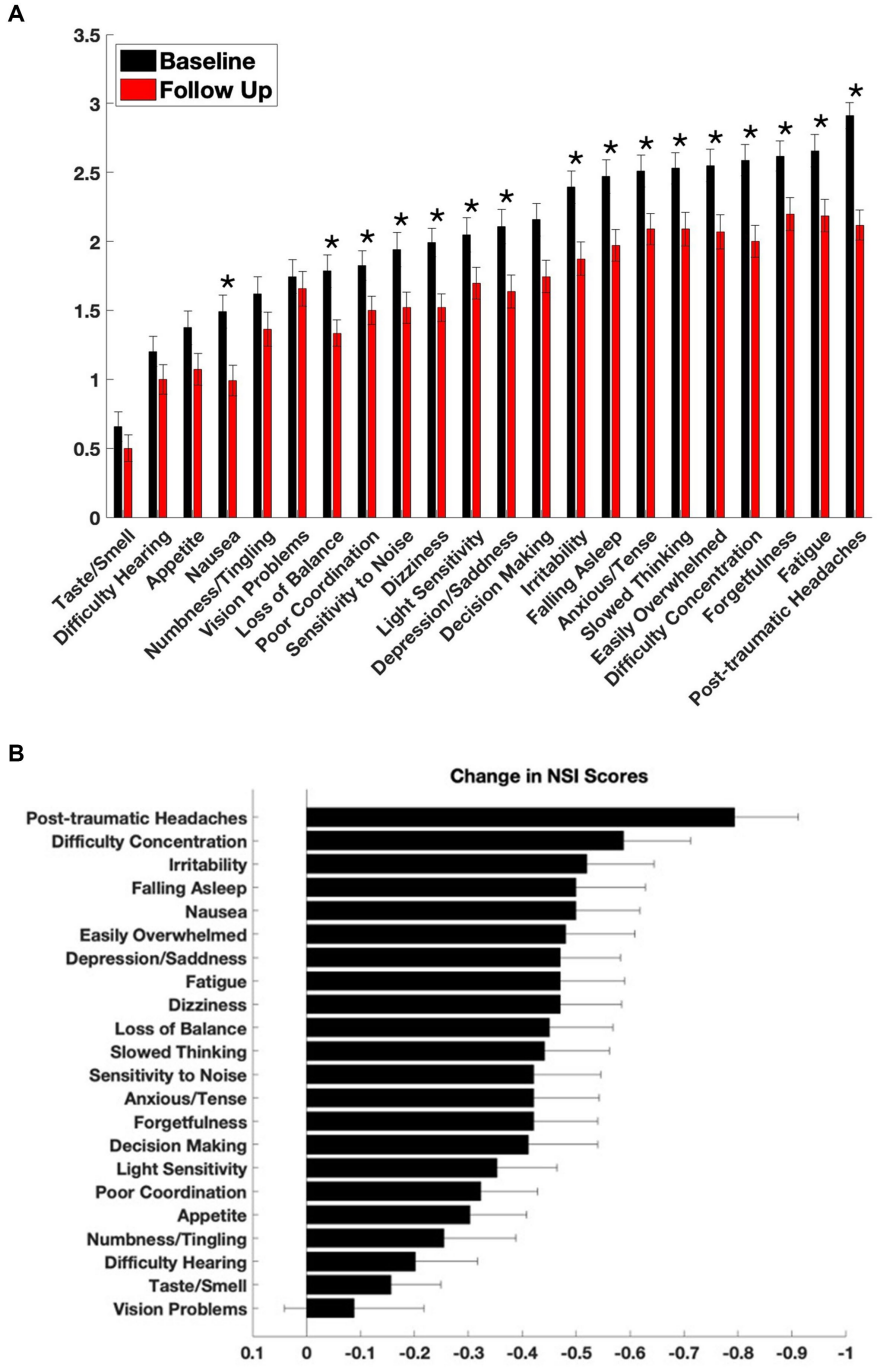
Symptom severity at baseline and after treatment with adjunctive nVNS. **(A)** Baseline and follow-up scores from the Neurobehavioral Symptom Inventory (NSI) are shown across 22 symptom domains. The NSI was administered at intake (black bars) and again approximately 110 days after initiation of non-invasive vagus nerve stimulation (nVNS) therapy (red bars). Each item is rated on a 0–4 scale, with higher scores indicating more severe symptoms. *By Wilcoxon signed-rank, *p* < 0.00227. **(B)** Mean change in NSI scores for each symptom, calculated as follow-up minus baseline scores for each patient. Negative values indicate improvement. Data are presented as mean ± standard error of the mean (SEM).

Sixteen out of twenty-two NSI symptom domains showed statistically significant improvement following 3 months of adjunctive nVNS combined with standard of care (SoC) treatment (Figure 1). The most significant reductions in symptom severity were observed for *post-traumatic headaches* (−0.79 ± 1.19; *p* = 1.97 × 10^−8^), *difficulty concentrating* (−0.59 ± 1.25; *p* = 1.79 × 10^−5^), *dizziness* (−0.47 ± 1.14; *p* = 7.11 × 10^−5^), and *depression/sadness* (−0.47 ± 1.12; *p* = 9.09 × 10^−5^).

Other symptoms with notable improvements included *nausea* (−0.50 ± 1.19; *p* = 0.00012), *difficulty falling asleep* (−0.50 ± 1.30; *p* = 0.00033), *irritability* (−0.52 ± 1.26; *p* = 0.00013), and feeling *easily overwhelmed* (−0.48 ± 1.30; *p* = 0.00027).

Four symptoms did not reach statistical significance: problems with *vision* (−0.09 ± 1.31; *p* = 0.35), *hearing* (−0.20 ± 1.16; *p* = 0.09), *numbness or tingling* (−0.25 ± 1.35; *p* = 0.07), and altered *taste or smell* (−0.16 ± 0.93; *p* = 0.10).

### Adjunctive nVNS supports clinically meaningful symptom improvement in mTBI

**Figure 2:**
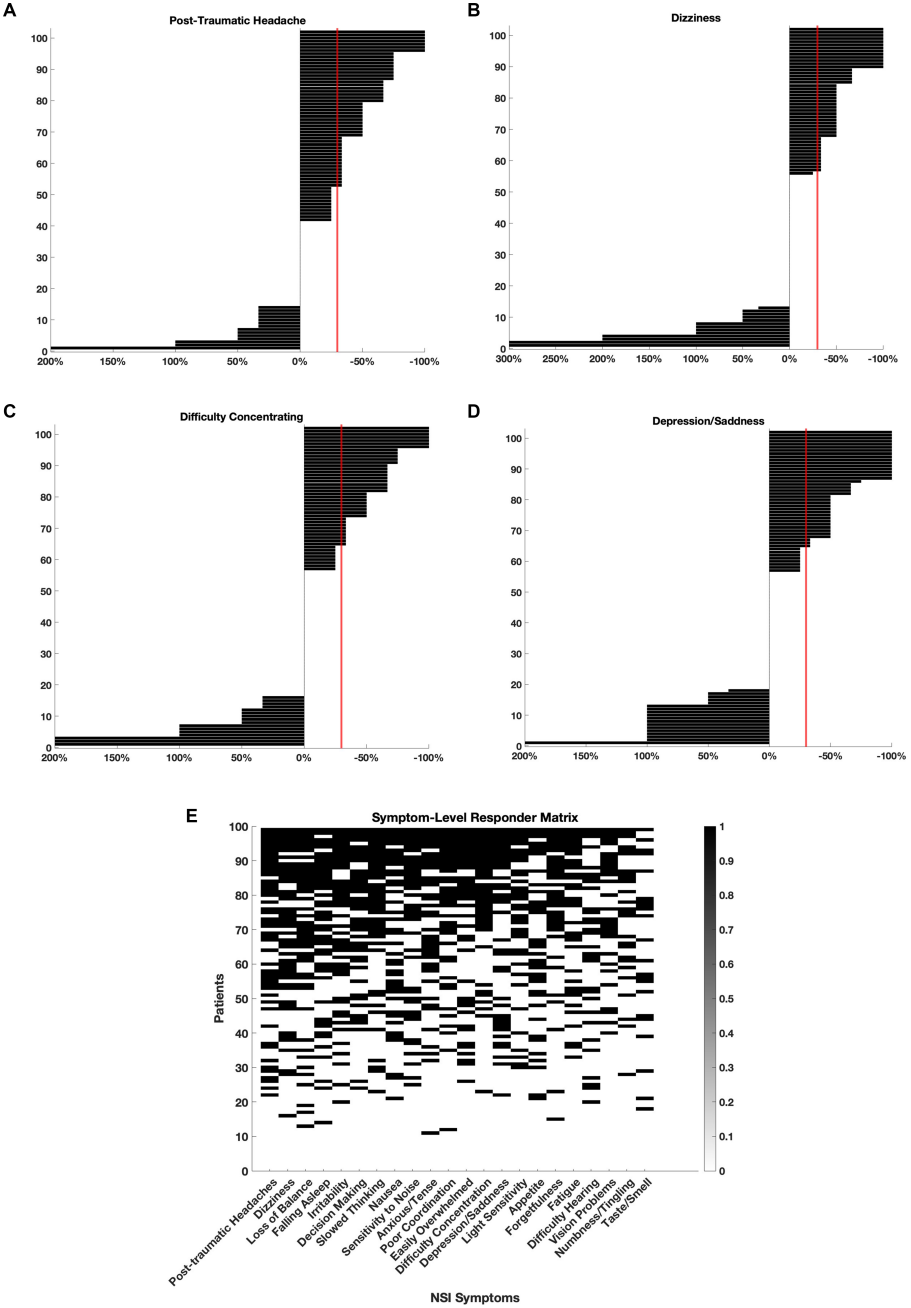
Clinically meaningful symptom improvements following adjunctive nVNS. For each symptom, individual patient-level changes in NSI scores were plotted to assess treatment response. Symptom improvement was calculated as the percent change from baseline, and patients who experienced a ≥ 30% reduction in severity (crossing the red line) were classified as ‘responders’. **(A)** *Post-traumatic headache*: 49% of patients (*n* = 50) were responders. **(B)** *Dizziness*: 45% of patients (*n* = 46) were responders. **(C)** *Difficulty concentrating*: 37% of patients (*n* = 38) were responders. **(D)** *Depression/sadness*: 37% of patients (*n* = 38) were responders. **(E)** Responder status for each patient and each symptom is represented in a binary matrix. Rows represent individual patients (*n* = 102), and columns represent NSI symptoms. A black square indicates that the patient met responder criteria for that symptom. Patients are sorted by total number of symptom responses (top to bottom), and symptoms are ordered by total number of responders (left to right).

To evaluate clinical relevance, we defined responders as patients who experienced a ≥ 30% reduction in NSI scores for specific symptom domains (Figure 2). Among the most significantly improved symptoms, 50 patients (49%) met responder criteria for *post-traumatic headache* (Figure 2A), 46 (45%) for *dizziness* (Figure 2B), 38 (37%) for *difficulty concentrating* (Figure 2C), and 38 (37%) for *depression/sadness* (Figure 2D).

In Figure 2E, the responder status for each patient and every symptom was plotted on a matrix. The symptoms were ordered left to right depending on the number of patients who met responder criteria. Over 90% of patients responded with at least 1 symptom and 34% of patients met responder criteria for half or more ( ≥ 11 out of 22) NSI domains. The symptoms with the most responders were *post-traumatic headache* (49%), *dizziness* (45%), *loss of balance* (45%), and *difficulty falling asleep* (44%) suggesting that nVNS supports the improvement of somatic and vestibular symptoms in particular across a heterogeneous mTBI population.

### The age of mTBI injury did not affect symptom severity at intake or treatment response

Age of injury may influence both the presentation of symptoms and the effectiveness of a given treatment. Although some symptoms may improve spontaneously, persistent post-concussive complaints can remain stable for months or even years, raising the question of whether treatment remains effective when initiated later in the post-injury course.

#### Severity of symptoms

**Figure 3:**
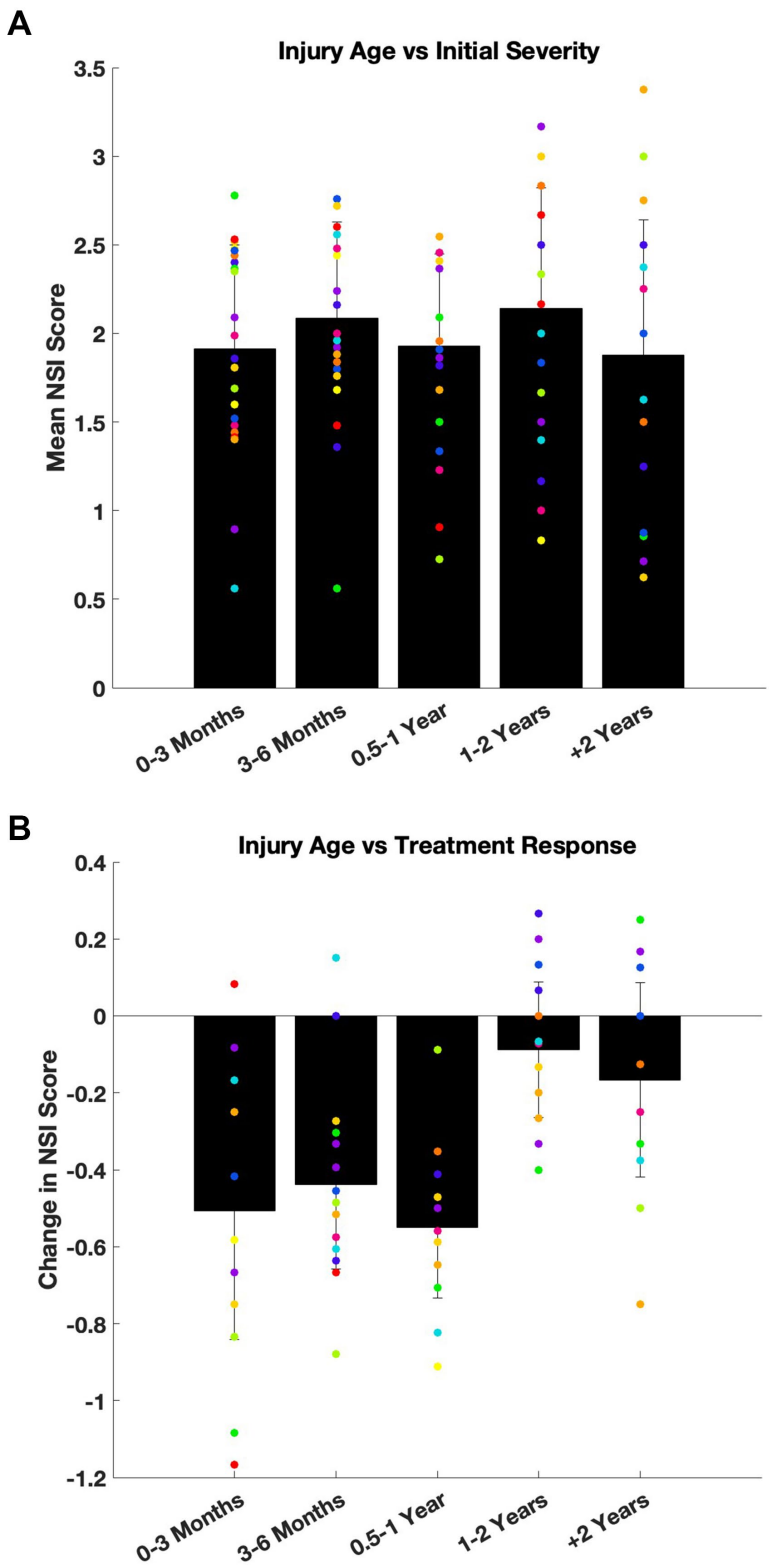
Age of injury did not affect baseline symptom severity or treatment response. **(A)** Baseline NSI scores were compared across five subgroups based on the age of injury: 0–3 months, 3–6 months, 6–12 months, 1–2 years, and more than 2 years old. Mean intake NSI scores are plotted with standard deviation (black bars: global severity across all 22 symptom; dots: average severity of each symptom). While one symptom domain (*appetite*) reached statistical significance by ANOVA (*p* = 0.047), no *post hoc* pairwise differences were detected. Overall, there was no consistent effect on severity of symptoms at intake due to the time since injury. **(B)** The same injury age subgrouping was used to evaluate treatment response, defined as change in NSI score (follow-up minus baseline). Mean change of NSI scores are shown with standard deviation (black bars: global change across all 22 symptom; dots: average change of each symptom). No statistically significant differences were observed across groups for treatment-related improvement (ANOVA, *p* > 0.05).

To assess whether time since injury influenced symptom presentation at intake, patients were stratified into five subgroups based on injury age at treatment initiation: <3 months (n = 77), 3–6 months (*n* = 25), 6–12 months (*n* = 22), 1–2 years (*n* = 6), and >2 years (*n* = 8). This analysis included 138 of the 175 patients with injury date and complete baseline NSI data. One symptom domain, *appetite*, showed a significant difference by ANOVA (*p* = 0.0472), but Scheffé’s *post hoc* analysis did not identify any pairwise differences. The remaining 21 parameters were not significant (*p* = 0.11–0.94). Figure 3A plots NSI severity at intake (black bar: global severity/mean of all 22 symptoms; dots: mean of individual symptoms) sorted by age of injury and demonstrates that there was no interaction between initial severity and time since injury.

To confirm that the absence of group differences was not due to limited statistical power, patients were also dichotomized into two groups: injury under 1 year old versus over 1 year old. Again, no effect of injury age on baseline symptom severity was observed (Mann–Whitney U tests; *p* = 0.10–0.98).

#### Efficacy of treatment

A similar analysis was performed to evaluate whether the age of injury influenced the response to treatment. This analysis included the 102 patients with complete baseline and follow-up NSI data. Symptom improvement was not significantly different across latency subgroups (ANOVA; *p* = 0.09–0.97; Figure 3B).

Taken together, these findings suggest that some patients whose concussion related symptoms do not resolve within 3 months may develop stable, persistent symptom profiles, and that adjunctive treatment with nVNS + SoC remains effective even when initiated months or years later.

#### Variability in NSI follow-up

To control of any effects due to the variability in timing for follow-up (112 ± 77 days), patients (*n* = 102) were again stratified into five subgroups based on the number of elapsed days since their intake and follow-up NSI surveys: 60 or fewer days (*n* = 28), 61–90 days (*n* = 22), 91–120 days (*n* = 16), 121–150 days (*n* = 15), and more than 150 days (*n* = 21). There was no interaction between symptom improvement and follow-up variability measured by ANOVA for any of the 22 symptoms (*p* = 0.10–0.97).

### Symptom severity predicts treatment response and aligns with secondary measures

**Figure 4:**
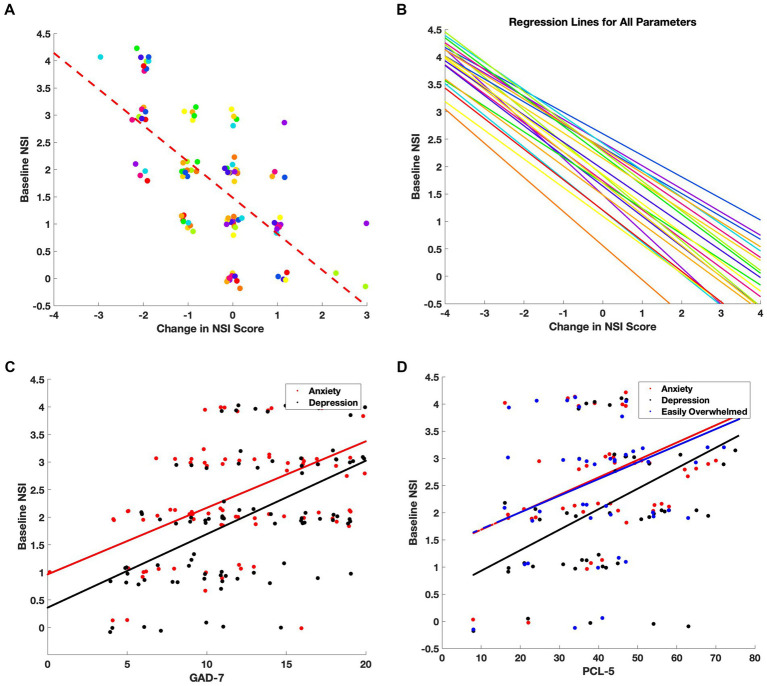
Baseline symptom severity predicts treatment-related improvement and correlates with clinical outcomes. **(A)** For each NSI item, individual patient scores were plotted as a function of baseline severity versus change in score (follow-up minus baseline). Points were jittered for clarity, and a linear regression line (dashed red) was fit. The strongest correlation was observed for the symptom: *Loss of Balance* (Pearson’s r = −0.67, R^2^ = 0.44). **(B)** Regression lines for all 22 NSI domains reveal a consistent negative slope, indicating that patients with higher baseline symptom severity tended to experience larger treatment-related improvements. **(C)** Correlations between baseline NSI scores for *Anxiety* (red) and *Depression/Sadness* (black) and GAD-7 scores. **(D)** Correlations between baseline NSI scores for *Anxiety* (red), *Depression/Sadness* (black), and *Feeling Easily Overwhelmed* (blue) and PCL-5 scores.

Pearson correlation analyses and linear regressions were conducted to explore relationships between baseline symptom severity, treatment response, and scores from additional survey instruments. A representative example is shown for *loss of balance*, which exhibited a strong negative correlation between initial severity and symptom improvement (Figure 4A; Pearson’s r = −0.67, R^2^ = 0.44). This relationship was consistent across all 22 NSI symptom domains (Figure 4B), indicating that patients with more severe symptoms at intake tended to experience greater improvement following therapy.

Correlations were also assessed between baseline NSI scores and the secondary questionnaires administered at intake. Notably, the NSI *anxiety* and *depression/sadness* items correlated strongly with GAD-7 scores (Figure 4C; r = 0.60 and r = 0.58, respectively), while *depression/sadness* also showed a robust association with PHQ-9 scores (r = 0.66), consistent with the intended use of those instruments. In addition, *depression/sadness* (r = 0.50), *anxiety* (r = 0.48), and *feeling easily overwhelmed* (r = 0.43) were positively correlated with PCL-5 scores (Figure 4D), suggesting alignment between NSI emotional symptom domains and PTSD-related distress.

## Discussion

In this retrospective observational study, we evaluated the clinical utility of adjunctive nVNS in patients with mTBI experiencing persistent post-concussive symptoms. Over a three-month treatment period, patients demonstrated statistically and clinically meaningful improvements across a broad range of symptom domains, with the most prominent effects observed for *post-traumatic headaches*, cognitive complaints (e.g., *difficulty concentrating*), vestibular symptoms (e.g., *dizziness*), and mood-related disturbances (e.g., *depression/sadness*). Over 1/3 of patients met responder criteria for 11 or more different symptoms. Importantly, these improvements occurred in a cohort with persistent symptoms and were independent of the time elapsed since injury, suggesting that nVNS + SoC may confer benefit even in cases of long-standing dysfunction.

### Clinical relevance and implications

These findings align with and extend prior evidence from controlled trials of nVNS in migraine and cluster headache, where neuromodulation has been shown to reduce pain severity, improve functional outcomes, and demonstrate sustained benefit across repeated attacks (24–26). Our results are also consistent with preclinical studies in rodent and mouse models of TBI, which demonstrate that nVNS can reduce lesion volume, suppress pro-inflammatory cytokine release, and improve behavioral performance (12, 16). Together, these findings support a translational continuum from mechanistic animal data to human clinical application in post-concussive care.

From a clinical perspective, the ease of administration, favorable tolerability profile, and multi-symptom efficacy of nVNS represent meaningful advantages in the management of mTBI, a condition with few targeted treatment options. While conventional approaches such as cognitive rehabilitation and pharmacotherapy are often limited by variable adherence and side effect burden (6, 22), nVNS may represent an additional non-pharmacologic therapeutic option that can safely be added to SoC and addresses both central and peripheral contributors to post-concussive pathology. The observed benefits across mood, sleep, vestibular, and cognitive domains underscore the potential for broad applicability in routine care.

No device-related adverse events were reported during the study period, consistent with prior trials demonstrating the safety and tolerability of nVNS across various headache and neurological indications (23, 27). The absence of systemic side effects and the device’s non-invasive nature make it particularly attractive for use in sensitive populations, including those with comorbid psychiatric or autonomic dysregulation.

A key exploratory analysis examined whether the latency from injury to treatment initiation influenced either baseline symptom severity or treatment response. Contrary to the concern that chronic symptoms might be refractory to intervention, we found no significant differences in symptom improvement across latency subgroups, including patients who initiated treatment more than 2 years post-injury. These findings are consistent with prior reports of symptom persistence and stabilization after initial injury (28) and suggest that therapeutic interventions remain relevant well beyond the acute or subacute period.

### Mechanistic interpretation

Although the precise mechanisms underlying the observed clinical improvements remain incompletely understood, several plausible biological pathways have been implicated. Stimulation of the cervical vagus nerve activates afferent projections to brainstem nuclei such as the nucleus tractus solitarius and locus coeruleus, which modulate both sympathetic tone and cortical excitability (17, 18). This activity has been shown to suppress systemic and central inflammation through the cholinergic anti-inflammatory reflex, with downstream effects on cytokine production, blood–brain barrier integrity, and neural network homeostasis (4, 10, 11). The strong correlation between baseline symptom severity and magnitude of improvement further supports a model in which nVNS may help normalize dysregulated circuits most affected by injury.

### Strengths, limitations, and future directions

The present study benefits from naturalistic clinical data, systematic symptom quantification using the NSI, and a relatively large cohort with persistent symptomatology. However, several limitations should be noted. The retrospective design precludes causal inference, and the absence of a control group introduces the potential for placebo effects and improvement due to personalized SoC procedures. While a non-nVNS comparator group was initially considered, baseline symptom differences suggested a fundamentally distinct population, limiting interpretability. Additionally, heterogeneity in comorbidities, treatment adherence, and adjunctive therapies may have introduced variability not captured in the available dataset.

These preliminary findings provide a strong rationale for prospective, controlled studies evaluating the use of nVNS in post-concussion populations. Future trials should aim to identify predictive biomarkers of response, optimize stimulation parameters, and evaluate durability of benefit. Given the observed multidimensional improvements, it may also be valuable to assess functional outcomes such as return to work, cognitive performance, or quality of life metrics in longitudinal designs.

## Conclusion

Adjunctive non-invasive vagus nerve stimulation was associated with clinically meaningful improvements in post-concussive symptoms across cognitive, affective, somatic, and vestibular domains in patients with mTBI. These findings build on a growing body of evidence supporting nVNS as a safe, mechanistically targeted, and broadly applicable intervention for neurologic dysfunction. Further controlled studies are warranted to establish efficacy and define its role in standard care.

## Data Availability

The raw data supporting the conclusions of this article will be made available by the authors, without undue reservation.
